# Epiplakin expression is lost in psoriatic skin lesions and is downregulated by IFN-γ in *ex vivo* skin cultures

**DOI:** 10.3389/fcell.2025.1617737

**Published:** 2025-07-17

**Authors:** Hannes Kühtreiber, Corinne Drexler, Melanie Salek, Lisa Auer, Johannes Griss, Michael Mildner, Peter Fuchs

**Affiliations:** ^1^ Department of Dermatology, Medical University of Vienna, Vienna, Austria; ^2^ Max Perutz Labs, Vienna Biocenter Campus (VBC), Vienna, Austria; ^3^ Max Perutz Labs, Department of Biochemistry and Cell Biology, University of Vienna, Vienna, Austria; ^4^ Applied Immunology Laboratory, Department of Thoracic Surgery, Medical University of Vienna, Vienna, Austria

**Keywords:** plakins, epiplakin, psoriasis, atopic dermatitis, scRNAseq

## Abstract

Proteins of the plakin family are predominantly expressed in the epidermis and play a crucial role in cytoskeletal assembly by crosslinking intracellular structural components with cell–cell junctions and the plasma membrane. While most plakins are critical for maintaining epidermal integrity, the role of epiplakin (EPPK1) in inflammatory skin disorders has not been thoroughly investigated. We therefore used single-cell RNA sequencing (scRNAseq) analysis, immunofluorescence, and *ex vivo* cytokine treatment of human skin explants to investigate EPPK1 regulation in psoriasis. ScRNAseq analysis of psoriatic and healthy skin revealed that *EPPK1* was the only member of the plakin family showing specific downregulation in the epidermis of psoriatic lesions. This finding was corroborated at the protein level by immunostaining of human psoriasis samples showing a specific downregulation of EPPK1 in the suprabasal granular layer of psoriatic epidermis. Transcriptomic profiling of Eppk1^−/−^ murine epidermis revealed reduced expression of genes involved in epithelial adhesion and lipid metabolism, partially overlapping with the psoriatic keratinocyte signature, suggesting that EPPK1 loss may predispose the skin to barrier dysfunction under inflammatory stress. Investigation of the mechanism underlying the EPPK1 regulation in psoriasis revealed that interferon-γ (IFN-γ) was the main cytokine involved in its downregulation in human *ex vivo* skin. Collectively, our findings demonstrate a specific IFN-γ-dependent downregulation of EPPK1 in psoriasis, suggesting that lack of EPPK1 might contribute to the epithelial defects observed in this inflammatory skin condition.

## 1 Introduction

Plakins are high-molecular-weight proteins which are also referred to as cytolinkers that play a crucial role in cytoskeletal assembly in various cells by crosslinking intracellular structural components with junctional complexes and the plasma membrane (Bouameur et al., 2014). The plakin family comprises seven members: plectin (*PLEC*), periplakin (*PPL*), microtubule-actin cross-linking factor 1 (*MACF1*), epiplakin (*EPPK1*), envoplakin (*EVPL*), desmoplakin (*DSP*), and bullous pemphigoid antigen 1 (*DST*) (Bouameur et al., 2014). These proteins are all expressed in the epidermis, and most have been demonstrated to be essential for epidermal integrity in knockout animal studies, with many members implicated in blistering skin diseases (Bouameur et al., 2014). One notable exception is EPPK1, the ablation of which did not result in any obvious alterations in the epidermal architecture in mice (Spazierer et al., 2006).

EPPK1 is distinct from all other plakins because it consists exclusively of plakin repeat domains (PRDs), whereas other plakin family members contain additional structural domains, such as actin and microtubule-binding domains (Spazierer et al., 2003; Bouameur et al., 2014). A detailed analysis of the *EPPK1* genomic locus in humans and mice has revealed the presence of multiple variants containing 9 to 16 PRDs, caused by varying numbers of identical PRD copies at the C-terminus (Ishikawa et al., 2018; Ueo et al., 2021). The expression of EPPK1 is restricted to epithelial tissues, with the highest levels found in the epidermis (Fujiwara et al., 2001; Spazierer et al., 2003). Co-localization of EPPK1 with keratin filaments has been demonstrated in cultured cell lines (Jang et al., 2005). Specifically, PRDs of mouse Eppk1 have been shown to bind to simple keratins 8 and 18, as well as epidermal keratins 5 and 14 (Spazierer et al., 2003; Szabo et al., 2015).

Interestingly, although the epidermis of *Eppk1* ^−/−^ mice did not show any ultrastructural abnormalities, faster keratinocyte migration was observed during wound healing, indicating a subtle role for Eppk1 in keratinocyte dynamics (Goto et al., 2006; Spazierer et al., 2006). A similar phenotype was observed in the cornea of *Eppk1* ^−/−^ animals, characterized by increased fragility, impaired intraepithelial cell differentiation, and accelerated cell migration of epithelial cells (Kokado et al., 2013). These findings suggest that while EPPK1 is not strictly required for epidermal integrity at least under non-stressed conditions, it may play a regulatory role in keratinocyte migration and wound repair. Recent live microscopy studies have shown that EPPK1 is diffusely distributed in the cytoplasm under homeostatic conditions, a finding that contrasts with previous reports describing its association with keratin filaments in fixed cells. It was further observed that under conditions of elevated cytoplasmic Ca^2+^ levels, EPPK1 associates with keratin filaments, leading to reduced keratin dynamics (Ratajczyk et al., 2022). In addition, EPPK1 presence in vertebrate genomes for over 450 million years, except in whales and dolphins, indicates an evolutionarily conserved role (Fuchs et al., 2022). However, it remains unclear whether EPPK1 mutations are associated with skin pathologies, as no reports have described human EPPK1 mutations linked to disease thus far.

As little is known about plakins in inflammatory skin conditions, we conducted the present study to investigate their expression and regulation in human and imiquimod induced murine psoriasis as well as atopic dermatitis, two chronic inflammatory skin diseases with different pathogenesis.

## 2 Materials and methods

### 2.1 Sample dissociation and preparation of the single-cell suspension

Wildtype and *Eppk1* ^*−/−*^ mice were bred and maintained as previously described (Spazierer et al., 2006). Mice were sacrificed, and dorsal skin was excised under sterile conditions. Tissue samples were washed in Dulbecco’s phosphate-buffered saline (PBS, without Ca^2+^/Mg^2+^; Gibco™, Thermo Fisher Scientific, Waltham, MA, United States, cat#: 14190144) under laminar airflow. Six mm punch biopsies were taken from intact dorsal skin of each a wild type C57BL/6J and a *Eppk1* ^−/−^ mouse backcrossed into the C57BL/6J background, mechanically minced, and enzymatically dissociated using the Whole Skin Dissociation Kit (Miltenyi Biotec, Bergisch Gladbach, Germany, cat#: 130-101-540) in gentleMACS™ C Tubes (Miltenyi Biotec, cat#: 130-093-237) for 2.5 h at 37°C. Dissociation was performed using the gentleMACS™ Octo Dissociator with Heaters (Miltenyi Biotec, cat#: 130-096-427), following the manufacturer’s instructions. This protocol yielded single-cell suspensions with >90% viability. Cell suspensions were filtered sequentially through 100 μm and 40 µm strainers (Corning Inc., Corning, NY, United States, cat#: 352360 and cat#: 352340, respectively), then washed twice with 0.04% bovine serum albumin (BSA; Sigma-Aldrich, St. Louis, MO, United States, cat#: A9418) in PBS. Cell concentration and viability were assessed using the Acridine Orange/Propidium Iodide (AO/PI) Cell Viability Kit (Logos Biosystems, Anyang-si, Gyeonggi-do, South Korea, cat#: F23001) and counted with the LUNA-FL™ Dual Fluorescence Cell Counter (Logos Biosystems, cat#: L20001).

### 2.2 Single-cell processing and library preparation

Single-cell RNA sequencing was performed using the Chromium Single Cell Fixed RNA Profiling Kit (10x Genomics, Pleasanton, CA, United States) according to the manufacturer’s instructions (CG000527, protocol version F). Approximately 10,000 cells per sample were targeted for recovery. Cells were hybridized with the Mouse Whole Transcriptome Probe Set (10x Genomics, Pleasanton, CA, United States, cat#: 1000496) and barcoded using the Chromium Next GEM Dual Index Kit TS Set A (10x Genomics, Pleasanton, CA, United States, cat#: 1000215) as part of the Pooled Wash Workflow. Library construction was performed using the Next GEM Single Cell Fixed RNA Hybridization and Library Kit (10x Genomics, Pleasanton, CA, United States, cat#: 1000415) and Gel Bead Kit (10x Genomics, Pleasanton, CA, United States, cat#: 1000421). Libraries were amplified using 9 cycles of PCR, followed by quality control using the Agilent 2100 Bioanalyzer (Agilent Technologies, Santa Clara, CA, United States), which showed clean profiles with expected fragment distributions.

### 2.3 Sequencing and data processing

Final libraries were sequenced on an Illumina NextSeq 2000 platform (Illumina Inc., San Diego, CA, United States) using a P4 100-cycle flow cell in paired-end mode, following 10x Genomics recommendations for the Fixed RNA Profiling workflow. Raw base call (BCL) files were demultiplexed using Cell Ranger mkfastq with the SI-TS-G2 dual index set (10x Genomics, Pleasanton, CA, United States). Reads were aligned to the mm10 mouse reference genome provided by 10x Genomics, and gene expression matrices were generated based on unique molecular identifier (UMI) counts.

### 2.4 scRNAseq analysis

Publicly available scRNAseq data sets GSE173706 (Ma et al., 2023), GSE165021,GSE150361,GSE193350 (Guo et al., 2024) and GSE153760 (Bangert et al., 2021) were retrieved from the NCBI Gene Expression Omnibus (GEO) database. Processing and analysis were performed using R (version 4.3.1, The R Foundation, Vienna, Austria) and RStudio (version 2023.6.2.0 + 561). Computational scRNAseq analyses were conducted using the Seurat package (version 4.9.9.9059) as per the developer’s vignettes (Hao et al., 2023). For each individual dataset, UMI count matrices were generated and converted to Seurat objects which were subsequently merged into a single object and subjected to quality control. For the psoriasis dataset (GSE173706) (Ma et al., 2023), cells were filtered based on the following criteria: fewer than 500 UMIs, fewer than 500 or more than 6,000 expressed genes, and more than 10% mitochondrial reads. Similarly, for the GSE153760 (Bangert et al., 2021) atopic dermatitis, the imiquimod dataset GSE165021,GSE150361,GSE193350 (Guo et al., 2024), as well as the data generated from *Eppk1* ^*−/−*^ mice (GSE300052) cells with fewer than 200 or more than 45,000 UMIs, fewer than 100 or more than 7,500 expressed genes, and more than 10% mitochondrial reads were excluded from further analysis. Additionally, all mitochondrial genes were removed from the UMI count matrices. Normalization and variance stabilization were performed using the ‘SCTransform’ function with v2 regularization, regressing out the percentage of mitochondrial reads. Dimensionality reduction was performed via principal component analysis (PCA) with 50 principal components, after which initial clustering was conducted using the ‘RunUMAP’, ‘FindNeighbors’, and ‘FindClusters’ functions. To correct for batch effects, the merged Seurat object was split by sample, and each subset was normalized. For data integration, the merged dataset was divided into ‘count’ and ‘data’ layers, followed by normalization and identification of variable features, per Seurat v5 integrative analysis guidelines. Features were scaled and centered before conducting PCA. Data integration was performed using the “IntegrateLayers” function with an anchor-based RPCA approach. The integrated, batch-corrected expression matrix was used for further analyses. Clustering was performed following the standard Seurat workflow, using UMAP and Louvain clustering with the “RunUMAP,” “FindNeighbors,” and “FindClusters” functions. Validation was performed by examining known marker panels to ensure the plausibility of initial clustering. For sub-clustering analysis, clusters of interest were subset, and the processes of normalization, variance stabilization, and integration were reiterated. PCA, UMAP, and Louvain clustering were then performed on the reintegrated subsets to derive sub-clusters. Differentially expressed genes (DEGs) were identified using the MAST statistical framework (Finak et al., 2015), implemented in Seurat’s ‘FindMarkers’ and ‘FindAllMarkers’ functions, focusing on genes expressed in a minimum of 25% of cells in one group. The “AddModuleScore” function within the Seurat environment was used for module score calculation of gene sets.

To functionally annotate DEGs, enrichment analysis was performed using the Enrichr R package (Kuleshov et al., 2016), querying the “GO_Biological_Process_2025”, “GO_Cellular_Component_2025”, and “WikiPathways_2024_Human” databases. Only significantly regulated genes (adjusted p-value < 0.05 and log_2_FC > 0.5) were included in the analysis. Enriched terms were ranked according to Enrichr’s “combined score,” which integrates the Fisher’s exact test p-value with the deviation from expected rank (z-score). The top enriched terms were visualized as dot plots indicating both combined score and gene ratio.

### 2.5 Data visualization

Data visualization was conducted using Seurat v. 4.9.9.9059 (Hao et al., 2023), ggplot2 v.3.4.2 (Wickham, 2016), EnhancedVolcano v.1.18.0 (Blighe et al., 2023), and scCustomize v.1.1.1 (Marsh et al., 2023).

### 2.6 Sampling of psoriatic and healthy skin

Lesional skin of psoriatic patients was collected at the Department of Dermatology, Medical University of Vienna via 6 mm punch biopsies. Healthy skin samples were obtained from abdominal tissue post plastic surgery at the Division of Plastic and Reconstructive Surgery, Department of Surgery, Medical University of Vienna.

### 2.7 *Ex-vivo* treatment of skin biopsies

The *ex-vivo* maintenance of the skin samples was carried out using KBM™-2 basal medium (Lonza Group AG, Basel, Switzerland, cat#: CC-3103) supplemented with KGM™-2 SingleQuots™ (Lonza Group AG, Basel, Switzerland, cat#: CC-4152), ensuring that the epidermis was not submerged in culture medium. Samples were treated with either 10 ng/mL recombinant IL-17A (R&D Systems®, cat#: 7955-IL/CF; n = 6), 10 ng/mL recombinant human IL-22 (R&D Systems®, cat#: 782-IL-010/CF; n = 4), 10 ng/mL recombinant human IL-1β (BioLegend®, cat#: 579402; n = 6), 10 ng/mL recombinant human IFN- γ (PeproTech®, cat#: 300-02-20UG; n = 6), a combination of the cytokines (n = 6), or medium alone (n = 6). Treatments were carried out for durations of 2 days, 4 days, or 8 days, with medium and cytokine changes performed every other day. After the treatment period, samples were fixed in 7.5% buffered neutral formaldehyde solution for further processing.

### 2.8 Immunofluorescence staining and microscopy

Paraffin-embedded sections from healthy donors, psoriasis patients, and *ex-vivo* treated skin samples were deparaffinized using a heater fan until the paraffin melted, followed by incubation in xylene. Sections were sequentially rehydrated through ethanol solutions (96%, 80%, 30%) and then rinsed in deionized water (dH2O). Antigen retrieval was performed using a 10 mM sodium citrate buffer (pH 6) prepared by diluting 25 mL of Target Retrieval Solution 10x (Dako, cat#: S2369) in 225 mL dH2O. Slides were placed in a jar containing 80 mL buffer and heated in a pressure cooker, followed by cooling to room temperature. The primary anti-EPPK1 antibody (Abcam, cat#: ab247172) was diluted 1:50 in 2% PBS/BSA, and 100 µL was added to each pap pen encircled section, followed by overnight incubation at 4°C. The sections were washed in PBS and incubated for 30 min at room temperature in the dark with Goat anti-Rabbit IgG (H+L), Alexa Fluor™ 546 (Invitrogen, ThermoFisher Scientific, cat#: A-11035), at a dilution of 1:500 in 2% PBS/BSA. The mixture included 10% goat serum and DAPI (1:1,000) (ThermoFisher Scientific, cat#: 62248). Sections were washed in PBS and dH2O and subsequently mounted with aqua polymount medium (Polysciences, cat#: 18606) and stored at 4°C. Fluorescence microscopy was conducted using an OLYMPUS BX63 fluorescence microscope, together with Olympus cellSens software (Olympus, Shinjuku, Tokyo, Japan).

### 2.9 Statistical analysis

Immunofluorescence quantification was performed using ImageJ (version 1.53t, National Institutes of Health, Bethesda, MD, United States). EPPK1 expression was assessed by calculating the Corrected Total Cell Fluorescence (CTCF), defined as: Integrated Density – (Area × Mean Background Fluorescence), providing a background-adjusted measure of total fluorescence signal over the EPPK1-positive area. All statistical analyses and data visualization were conducted using GraphPad Prism version 10.0.3 (GraphPad Software, San Diego, CA, United States). Comparisons involving more than two groups were analyzed using one-way ANOVA followed by Dunnett’s multiple comparisons test. Differences between two groups were assessed using unpaired two-tailed *t*-tests. Associations between continuous variables were examined using Pearson’s correlation coefficient. Relationships between categorical and continuous variables were analyzed by simple logistic regression. A *p*-value < 0.05 was considered statistically significant. For multiple comparisons, adjusted *p*-values were applied where indicated.

## 3 Results

### 3.1 *EPPK1* mRNA-expression is significantly reduced in psoriatic lesions

**FIGURE 1:**
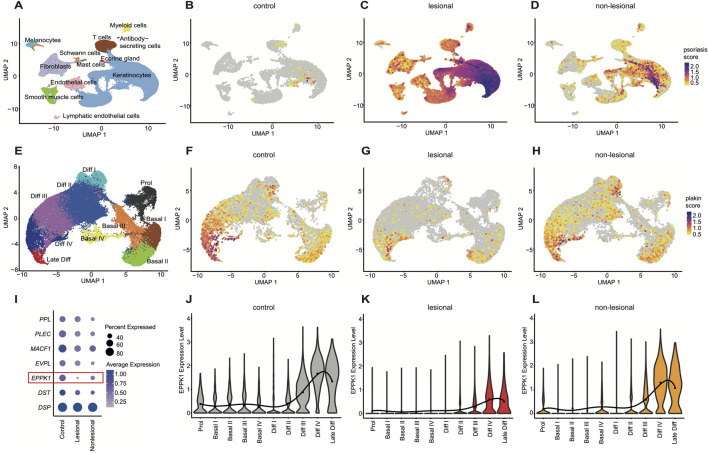
Comprehensive scRNAseq analysis of plakin regulation in psoriasis **(A)** UMAP visualization from a comprehensive scRNAseq analysis, revealing 12 distinct cell clusters. **(B–D)** Feature plots illustrating a psoriasis module score, including the expression levels of *S100A7A, S100A8, S100A9, IL17A, IL1B, IL22, IFNG, KRT6A, KRT16, MKI67*, and *IL23A* genes. Feature plots are split by condition into **(B)** (F) control, **(C)** lesional, and **(D)** non-lesional psoriasis. **(E)** UMAP of a subset from the keratinocyte cluster, showing 10 distinct clusters representing stages of keratinocyte differentiation. **(F–H)** Feature plots showing a plakin module score, including expression levels of *DSP, DST, EPPK1, EVPL, MACF1, PLEC*, and *PPL* genes, split by condition into **(F)** control, **(G)** lesional, and **(H)** non-lesional. **(I)** Dot plot showing expression levels of epidermal plakins in the keratinocyte subset split by condition. Dot size represents the expression percentage for each gene with color intensity reflecting average gene expression levels. **(J–L)** *EPPK1* expression levels as violin plots, split by condition. The mean expression level for each keratinocyte cluster is indicated by a black dot, with the solid black line representing a smooth sixth-degree polynomial regression fit through the means. **(J)** Grey violins show *EPPK1* expression levels in healthy skin, **(K)** red violins illustrate *EPPK1* expression in lesional psoriasis, and **(L)** orange violins show *EPPK1* expression in non-lesional psoriasis.

To assess the expression levels of plakin family members, we re-analyzed a previously published scRNAseq dataset (Ma et al., 2023) from lesional and non-lesional psoriatic skin, alongside healthy controls. Following initial clustering of cell types (Figure 1A; Supplementary Figure S1A), we calculated a psoriasis module score based on key psoriasis-associated genes (*S100A7A*, *S100A8*, *S100A9*, *IL17A*, *IL1B*, *IL22*, *IFNG*, *KRT6A*, *KRT16*, *MKI67*, and *IL23A*), which revealed a strong increase in lesional psoriatic skin and a moderate increase in non-lesional skin compared to healthy controls (Figures 1B–D), confirming accuracy of the disease-state and providing a reliable platform for further analysis. Notably, the module score also incorporated proinflammatory cytokines (*IL17A*, *IL1B*, *IL22*, *IL23*, and *IFNG*) which were upregulated in lesional psoriasis, providing context for the regulation of plakin family members (Figures 1B–D). Subsequent keratinocyte subsetting (Figure 1E) and clustering into differentiation stages, using well-established marker genes (Supplementary Figures S1B, S2), identified proliferative basal keratinocytes and differentiated keratinocyte stages. A plakin gene module score, derived from the expression levels of all plakin members, including *DSP*, *DST*, *EPPK1*, *EVPL*, *MACF1*, *PLEC*, and *PPL*, was subsequently calculated. This revealed a marked reduction in the overall expression of plakin family members in lesional psoriatic skin (Figures 1F–H), while expression levels in non-lesional skin remained largely similar to healthy controls. Further dissection of individual plakin family members expression revealed that *EPPK1* stood out as the most significantly downregulated plakin in psoriasis (Figure 1I). Violin plots illustrated that *EPPK1* expression was substantially decreased across all epidermal layers of lesional skin, with a pronounced reduction observed in the late differentiation stages of keratinocytes (Figures 1J–L). Non-lesional psoriatic skin showed a moderate reduction in *EPPK1* expression compared to lesional skin, though its levels remained lower than those observed in healthy controls. Interestingly, all other plakin family members showed less, or no deviating expression levels in psoriasis (Supplementary Figure S3). Importantly, the significant downregulation of *EPPK1* contrasts with the relatively stable expression of *EVPL* and *PPL*, two plakins highly expressed in differentiated keratinocytes (Figure 1I; Supplementary Figure S3). Taken together with the reduced but largely preserved expression of key terminal differentiation markers *FLG* and *CDSN* (Supplementary Figures S2E, F), this pattern indicates that *EPPK1* downregulation is unlikely to be solely a secondary consequence of impaired keratinocyte differentiation in lesional psoriasis. These findings underscore the distinct downregulation of *EPPK1* mRNA expression in psoriasis, particularly in relation to keratinocyte differentiation, with non-lesional skin retaining *EPPK1* levels closer to healthy controls.

### 3.2 *Eppk1^−/−^* keratinocytes downregulate adhesion and lipid genes, partly mirroring psoriatic profiles

**FIGURE 2:**
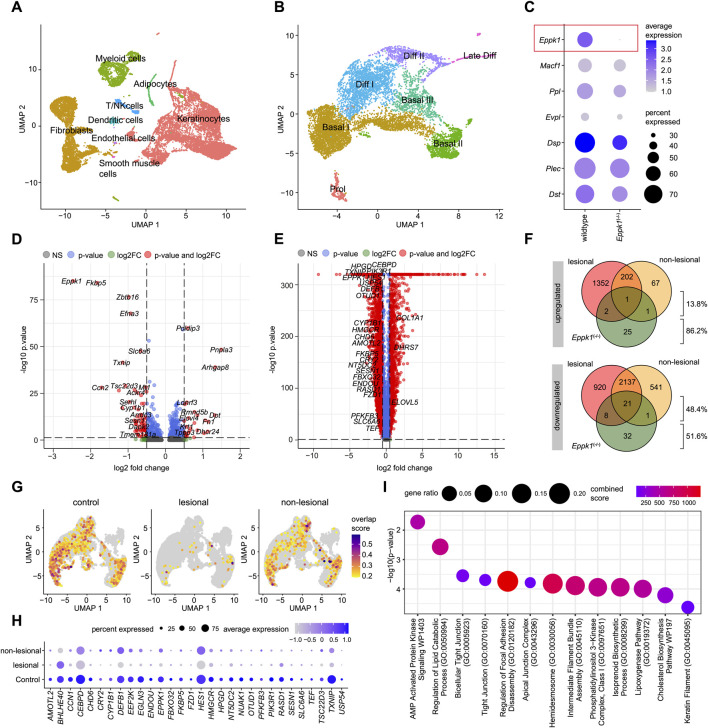
Comparative scRNA-seq analysis of *Eppk1^−/−^* and psoriatic keratinocytes **(A)** UMAP visualization of single-cell RNA-seq data from murine wildtype and *Eppk1* ^−/−^ skin, revealing 8 distinct clusters. **(B)** UMAP of the keratinocyte subset showing seven subclusters corresponding to stages of keratinocyte differentiation. **(C)** Dot plot displaying expression of plakin family genes, split by condition into wildtype and *Eppk1* ^−/−^. Dot size reflects the percentage of expressing cells, and color intensity indicates average expression levels. **(D)** Volcano plot showing differentially expressed genes (DEGs) between wildtype and *Eppk1* ^−/−^ keratinocytes. Genes significantly upregulated in *Eppk1* ^−/−^ (log_2_FC > 0.5, adjusted p < 0.05) are shown with positive fold changes, while downregulated genes exhibit negative fold changes. **(E)** Volcano plot of DEGs between healthy skin and lesional psoriasis within the keratinocyte subset (based on data shown in Figure 1). Significantly upregulated genes in psoriasis compared to healthy skin have positive log_2_FC values, and significantly downregulated genes have negative log_2_FC values. **(F)** Venn diagrams illustrating the overlap of significantly regulated genes in keratinocytes between *Eppk1* ^−/−^, lesional psoriasis, and non-lesional psoriasis, compared to healthy controls/wildtype. **(G)** Feature plot showing a module score for genes that are consistently downregulated across *Eppk1* ^−/−^ and human psoriasis datasets (as identified in F), split by condition. **(H)** Dot plot of individual overlapping downregulated genes (from F) across conditions in the human psoriasis dataset. Dot size reflects the percentage of expressing cells; color intensity reflects average expression. **(I)** Enrichment analysis of overlapping downregulated genes in *Eppk1* ^−/−^ and psoriatic keratinocytes. Top 13 enriched pathways are shown with corresponding combined scores and gene ratios.

To investigate the role of EPPK1 in epidermal homeostasis and its potential involvement in psoriasis-associated transcriptional changes, we performed scRNA-seq of skin isolated from wildtype and *Eppk1* ^−/−^ mice. Following initial clustering of epidermal populations (Figure 2A), we subsetted the keratinocyte compartment (Figure 2B) and assessed the expression of plakin family members. As expected, *Eppk1* expression was selectively lost in knockout keratinocytes. In contrast, expression of other plakins, including *Macf1*, *Ppl*, *Evpl*, *Dsp*, *Plec*, and *Dst* remained largely unchanged between wildtype and *Eppk1* ^−/−^ mice (Figure 2C), indicating that loss of *Eppk1* is not compensated by transcriptional upregulation of related plakin genes. Differential gene expression analysis within the keratinocyte subset identified 29 significantly upregulated and 62 significantly downregulated genes in *Eppk1* ^−/−^ skin compared to wildtype controls (log_2_FC > 0.5, adjusted *p* < 0.05; Figure 2D). This transcriptional shift was relatively modest compared to the broader dysregulation observed in lesional psoriatic keratinocytes versus healthy skin (Figure 2E). Strikingly, however, nearly half (48.4%) of the significantly downregulated genes in *Eppk1* ^−/−^ keratinocytes were also suppressed in lesional psoriatic keratinocytes (Figure 2F), suggesting transcriptional convergence in epidermal dysregulation. Visualization of a module score based on the overlapping downregulated gene set revealed pronounced suppression in lesional keratinocytes, with an intermediate reduction in non-lesional psoriasis (Figure 2G), a pattern that was also evident at the individual gene level (Figure 2H). Enrichment analysis revealed that the shared downregulated genes are significantly associated with pathways essential to epidermal function, including tight and apical junction assembly, hemidesmosome organization, focal adhesion dynamics, intermediate filament bundling, and lipid metabolic processes (Figure 2I). These data indicate that *Eppk1* loss partially recapitulates psoriasis-like transcriptional changes in keratinocytes and suggest a role for *Eppk1* in maintaining epidermal integrity, promoting barrier formation, and supporting resilience during stress.

### 3.3 IMQ-induced psoriasis mouse model exhibits generalized downregulation of plakins including *Eppk1*

**FIGURE 3:**
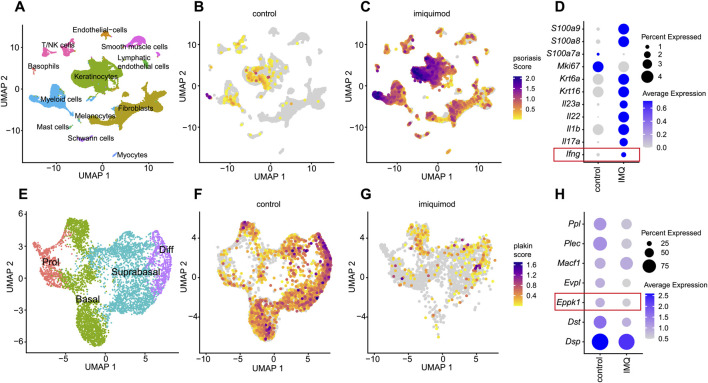
Comprehensive scRNAseq analysis of plakin regulation in IMQ-induced murine psoriasis **(A)** UMAP visualization from a scRNAseq analysis, revealing 12 distinct cell clusters. **(B,C)** Feature plots illustrate a psoriasis module score, including the expression levels of *Ifng, Il17a, Il1b, Il22, Il23a, Krt16, Krt6a, Mki67, S100a7a, S100a8, S100a9* genes. Feature plots are split by condition into **(B)** healthy skin and **(C)** imiquimod (IMQ) conditions. **(D)** Dot plot of psoriasis related genes split by condition. Dot size represents the expression percentage for each gene with color intensity reflecting average gene expression levels. **(E)** UMAP of a subset from the keratinocyte cluster, showing 4 clusters representing stages of keratinocyte differentiation. **(F,G)** Feature plots showing a plakin module score, including expression levels of *Dsp, Dst, Eppk1, Evpl, Macf1, Plec*, and *Ppl* genes, split by condition into **(F)** healthy controls and **(G)** imiquimod (IMQ). **(H)** Dot plot of epidermal plakins in the keratinocyte subset split by condition. Dot size represents the expression percentage for each gene with color intensity reflecting average gene expression levels.

To explore whether *EPPK1* downregulation is conserved in mouse models of psoriasis, we performed scRNA-seq analysis of skin from wild-type and imiquimod (IMQ)-treated mice (Guo et al., 2024). Unsupervised clustering identified 12 cell populations (Figure 3A), with cluster identities defined by established marker gene expression (Supplementary Figure S1D). Consistent with findings in human psoriasis, the psoriasis-associated gene module score was markedly elevated in IMQ-treated skin compared to controls (Figures 3B,C), confirming disease induction. Most genes within the module were upregulated in the IMQ condition, including *Il23a*, *Il17a*, *Il22*, *Il1b*, *Ifng*, and *S100a8/9*, whereas *Mki67* exhibited higher expression in control samples (Figure 3D), possibly reflecting reduced proliferative drive or differing kinetics of proliferation in the IMQ model compared to human psoriasis. Focusing on keratinocytes, we subsetted and reclustered this population into four differentiation stages (Figure 3E), corroborated through canonical marker genes (Supplementary Figure S1E). Calculation of a plakin gene module score, comprising *Dsp*, *Dst*, *Eppk1*, *Evpl*, *Macf1*, *Plec*, and *Ppl*, revealed a broad reduction in the IMQ-treated condition, partially recapitulating the findings observed in human lesional psoriasis (Figures 3F,G). Interestingly, whereas *Eppk1* was markedly and specifically downregulated in human lesional psoriatic skin, the IMQ mouse model exhibited a broader and less selectively reduced expression of plakins, including *Ppl*, *Evpl*, *Plec*, and *Dst* (Figure 3H). These differences might indicate species-specific responses or distinct inflammatory mechanisms underlying epidermal structural disruptions in the IMQ model compared to human psoriasis.

### 3.4 *EPPK1* shows consistent expression in atopic dermatitis

**FIGURE 4:**
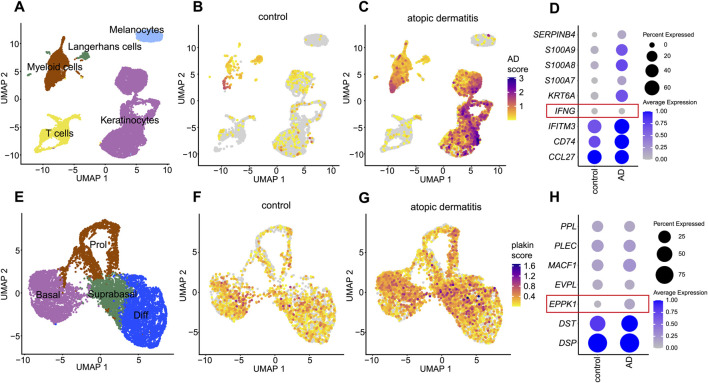
Comprehensive scRNAseq analysis of plakin regulation in atopic dermatitis **(A)** UMAP visualization from a scRNAseq analysis, revealing 5 distinct cell clusters. **(B,C)** Feature plots illustrate an atopic dermatitis module score, including the expression levels of *CCL27, CD74, IFITM3, IFNG, KRT6A, S100A7, S100A8, S100A9* and *SERPINB4* genes. Feature plots are split by condition into **(B)** healthy skin and **(C)** atopic dermatitis (AD). **(D)** Dot plot of AD-related genes split by condition. Dot size represents the expression percentage for each gene with color intensity reflecting average gene expression levels. **(E)** UMAP of a subset from the keratinocyte cluster, showing 4 clusters representing stages of keratinocyte differentiation. **(F,G)** Feature plots showing a plakin module score, including expression levels of *DSP, DST, EPPK1, EVPL, MACF1, PLEC*, and *PPL* genes, split by condition into **(F)** healthy skin and **(G)** atopic dermatitis (AD). **(H)** Dot plot of epidermal plakins in the keratinocyte subset split by condition. Dot size represents the expression percentage for each gene with color intensity reflecting average gene expression levels.

To evaluate whether the observed downregulation of *EPPK1* is specific to psoriasis or also occurs in other chronic inflammatory skin diseases, we performed a comparative analysis using atopic dermatitis (AD) another chronic inflammatory skin disorder to contextualize the psoriasis findings. We thus re-analyzed our previously published dataset (Bangert et al., 2021) to assess plakin expression in skin lesions of AD. Initial clustering of cell types identified five distinct clusters, including keratinocytes, melanocytes, T cells, Langerhans cells, and myeloid cells (Figure 4A). An AD module score was then calculated based on the expression levels of key AD-related genes (*CCL27*, *CD74*, *IFITM3*, *IFNG*, *KRT6A*, *S100A7*, *S100A8*, *S100A9*, and *SERPINB4*), revealing a clear distinction between control and AD samples (Figures 4B,C). Notably, our analysis revealed that in contrast to psoriasis, *IFNG* expression showed little to no variation between healthy skin and AD (Figure 4D), whereas increased levels of *IFNG* have been observed in psoriasis, particularly in lesional skin within the T-cell cluster (Supplementary Figure S4). Next, keratinocytes were subsetted and clustered into four distinct differentiation stages: proliferative, basal, differentiating, and late differentiating keratinocytes (Figure 4E). A plakin gene module score was calculated to assess overall plakin expression, revealing only minor differences between AD and control samples, with a slight overall increase in plakin expression observed in AD (Figures 4F,G). Upon further analysis of individual plakin family members, little or no difference in expression levels was found between AD and control conditions. Interestingly, *EPPK1* exhibited no marked differences in mRNA expression between AD and controls, with a slight increase observed in AD, contrasting with the significant downregulation as seen in psoriatic lesions (Figure 4H). These findings suggest that while minor alterations in overall plakin expression were observed in AD, with a slight increase in the plakin module score, reduced *EPPK1* expression appears to be more characteristic of psoriasis.

### 3.5 Immunostaining confirms EPPK1 downregulation in psoriatic lesions

**FIGURE 5:**
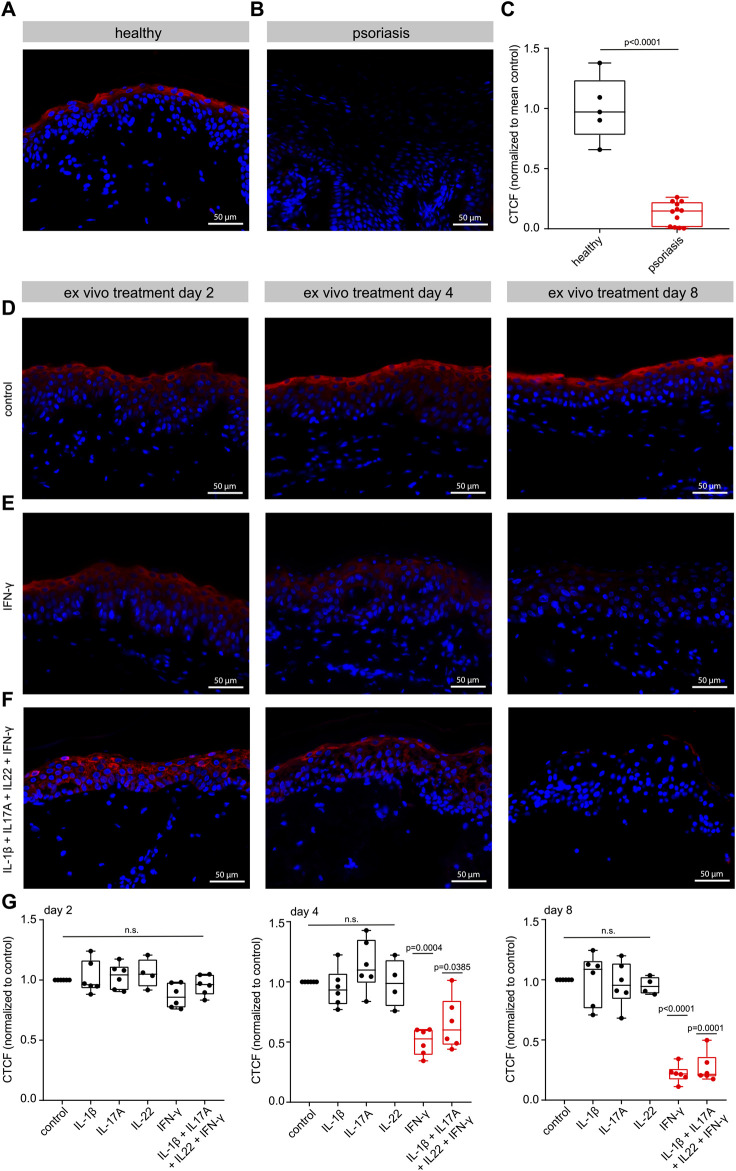
EPPK1 immunostaining and *ex-vivo* cytokine treatment. Immunofluorescence imaging of **(A)** healthy skin and **(B)** psoriatic skin. Images at ×40 magnification display DAPI-stained nuclei (blue) and EPPK1 fluorescence (red). **(C)** Quantification of EPPK1 signal intensity based on corrected total cell fluorescence (CTCF) normalized to the mean of healthy control samples. Data are presented as box plots displaying all individual data points. Red indicates statistically significant differences (p < 0.05). **(D–F)** Immunofluorescence staining of human skin cultured *ex vivo* and treated with pro-inflammatory cytokines. Representative images at ×40 magnification showing EPPK1 (red) and DAPI (blue) at days 2, 4, and 8 post-treatment. **(D)** Untreated *ex vivo* skin. **(E)** IFN-γ treatment. **(F)** Combined treatment with IL-1β, IL-17A, IL-22, and IFN-γ. **(G)** Quantification of EPPK1 expression (CTCF) in cytokine-treated *ex vivo* skin, normalized to untreated control samples. Data are shown as box plots with all data points. Red highlights statistically significant changes (adjusted p < 0.05).

Consequently, we further concentrated our study on EPPK1 in psoriasis, using immunostaining to validate the mRNA expression results from the scRNAseq analysis. Immunofluorescence staining in healthy skin revealed strong EPPK1 protein expression, specifically localized to the suprabasal granular layer of the epidermis, particularly in late-stage differentiated keratinocytes (Figure 5A). This pattern is consistent with the elevated expression levels observed in the “Diff III”, “Diff IV”, and “Late Diff” keratinocyte clusters from the scRNA-seq analysis (Figures 1F,J). In sharp contrast, virtually no EPPK1 staining was observed in lesional psoriatic skin (Figure 5B), consistent with the significant downregulation of *EPPK1* mRNA observed in our earlier transcriptomic analysis (Figures 1G,K). In line with these observations, semi-quantitative analysis revealed a significantly lower corrected total cell fluorescence (CTCF) of EPPK1 in psoriatic lesions compared to healthy skin (p < 0.0001; Figure 5C). These findings were corroborated across all psoriasis donors analyzed (n = 12), with a marked absence of EPPK1 staining in the majority of cases, while EPPK1 expression was consistently detected in healthy skin samples (Supplementary Figure S5). Interestingly, EPPK1 staining intensity in psoriatic lesions showed no significant association with sex (Tjur’s R^2^ = 0.022, p = 0.6204), disease duration (R^2^ = 0.2025, p = 0.1421), or PASI scores (R^2^ = 0.0039, p = 0.8477), indicating that EPPK1 downregulation occurs independently of these clinical parameters (Supplementary Table S1; Supplementary Figure S6).

### 3.6 IFN-γ mediates EPPK1 downregulation in *ex-vivo* skin cultures

We further aimed to investigate the factors responsible for the observed downregulation of EPPK1 in psoriasis. To this end, we first examined the expression of *IL1B*, *IL17*, *IL22* and *IFNG*, all well-known cytokines involved in the pathogenesis of psoriasis (Baliwag et al., 2015; He et al., 2023), in the scRNAseq dataset. As shown in Supplementary Figure S4, *IL17A*, *IL22* and *IFNG* were strongly upregulated in specific subsets of T-cells, and *IL1B* expression was upregulated in myeloid cells. To explore the effects of these cytokines on EPPK1 expression, we stimulated biopsies of healthy skin *ex vivo* with each of these cytokines individually and in combination. After 2, 4, and 8 days, we harvested the samples and performed immunostaining for EPPK1 (Figures 5D–F). While stimulation with IL-1β, IL-17A, or IL-22 alone did not significantly alter EPPK1 expression in this setting (Supplementary Figure S7), treatment with IFN-γ led to a marked reduction in EPPK1 levels by day 4 (p = 0.0385) and an even more pronounced decrease by day 8 (p < 0.0001) (Figures 5E,G). Notably, IFN-γ–treated samples also displayed mild parakeratosis, reminiscent of psoriatic histopathology. Combined treatment with IL-1β, IL-17A, IL-22, and IFN-γ produced similar effects to IFN-γ alone (Figures 5F,G), suggesting that IFN-γ is the dominant effector in this cytokine mix. Quantitative analysis of EPPK1 CTCF corroborated these observations, showing statistically significant reductions in EPPK1 intensity upon IFN-γ exposure and combinatorial treatment (Figure 5G). These findings indicate that IFN-γ is a key negative regulator of EPPK1 expression in keratinocytes and may contribute to its downregulation in psoriatic lesions.

## 4 Discussion

In the present study we investigated the role of EPPK1 in inflammatory skin conditions. Our data demonstrate that EPPK1 is robustly expressed in the suprabasal granular layer of healthy human epidermis, yet markedly reduced at both the mRNA and protein levels in lesional psoriatic skin. This finding contrasts with previous reports suggesting increased EPPK1 expression in psoriasis (Tian et al., 2022). A plausible explanation for this discrepancy may lie in methodological differences, including antibody specificity and the use of immunohistochemistry in earlier studies versus scRNA-seq and immunofluorescence in our work. Notably, we found that *EPPK1* shares an expression pattern with other plakins such as *EVPL* and *PPL*, both of which are well-established markers of epidermal differentiation in healthy skin. However, unlike *EPPK1*, *EVPL* and *PPL* were not downregulated in lesional psoriasis, indicating a unique role of *EPPK1* in psoriatic pathogenesis.

Structurally, EPPK1 stands out among plakins due to its lack of a coiled-coil rod domain, functioning likely as a single-chain molecule (Fujiwara et al., 2001; Bouameur et al., 2014). Its conservation across nearly all vertebrate species, with the notable exception of cetaceans, emphasizes its evolutionary importance (Fuchs et al., 2022). The absence of EPPK1 in cetaceans may be attributed to their aquatic environment, which imposes lower mechanical stress on the skin. Interestingly, cetacean skin exhibits morphological parallels to psoriatic epidermis, including thickening, hyperkeratosis, and parakeratosis (Springer et al., 2021), raising intriguing questions about the evolutionary role of EPPK1 in barrier adaptation.

Given that EPPK1 protein expression is most prominent in late-stage differentiated keratinocytes, its reduction in psoriasis may contribute primarily to disrupted differentiation rather than the hyperproliferation that characterizes the disease. An effect on cell proliferation has previously been reported in cancer models showing that EPPK1 knockdown inhibits cell proliferation (Yang et al., 2023; Arimura et al., 2024), a phenotype opposite to that observed in psoriatic epidermis. The apparent contradiction could reflect context-specific differences in the drivers of proliferation. In psoriasis, chronic inflammation, particularly mediated by IFN-γ, IL-17A, and IL-22 (Niehues et al., 2021), may override any anti-proliferative effect of EPPK1 loss. Therefore, we propose that EPPK1 downregulation predominantly impairs keratinocyte differentiation and migration (Goto et al., 2006). However, it remains unclear whether EPPK1 contributes directly to the barrier defect observed in psoriasis or if its downregulation is merely a bystander effect of impaired keratinocyte differentiation. Importantly, several lines of evidence argue against the idea that EPPK1 downregulation is a mere secondary consequence of altered differentiation. For example, terminal differentiation markers like *FLG* and *CDSN* remain largely intact in psoriatic skin, and other suprabasal plakins with similar expression profiles are unaffected. Furthermore, we found that IFN-γ stimulation of *ex vivo* skin samples led to a significant reduction in EPPK1 expression within 4 days, without impacting overall skin architecture. This suggests that the regulation of EPPK1 is cytokine-driven and occurs independently of gross differentiation defects. Thus, IFN-γ may act as a direct regulator of EPPK1 in the context of inflammation. Nonetheless, additional research is required to definitively address this issue.

Our exploration of Eppk1-deficient mice revealed no overt epidermal abnormalities under steady-state conditions (Spazierer et al., 2006), reinforcing the notion that EPPK1 is not essential for maintaining basic epidermal morphology. However, scRNA-seq of *Eppk1* ^*−/−*^ murine skin revealed reduced expression of genes associated with epithelial cohesion and lipid barrier formation. Intriguingly, nearly half of these genes were also downregulated in human psoriatic keratinocytes, pointing to a shared transcriptional program that may undermine epidermal homeostasis and barrier function. While Eppk1 deficiency alone does not cause spontaneous skin pathology, the overlapping gene signature may sensitize the skin to inflammation-induced damage. Notably, *Eppk1* ^*−/−*^ keratinocytes did not exhibit compensatory upregulation of other plakin family members (*Ppl*, *Evpl*, *Dst*, etc.), key cytoskeletal genes such as *Krt5*, *Krt14*, or actin-associated linker proteins. A similar absence of compensation was observed in lesional psoriatic keratinocytes, suggesting that EPPK1 loss creates a structural void that is not buffered by alternative cytolinker proteins. This aligns with previous work in plectin-deficient cells, where keratin filament instability was not corrected by transcriptional adaptation but was instead shaped by post-translational mechanisms and MAPK signaling (Osmanagic-Myers et al., 2006). Similarly, EPPK1 loss does not appear to trigger a compensatory transcriptional program, though compensation at the post-transcriptional level, possibly involving ERM proteins or filamins, remains a possibility and warrants further study.

In support of our human findings, the IMQ-induced mouse model of psoriasis also showed a downregulation of *Eppk1*. However, in contrast to the selective downregulation observed in human psoriasis, IMQ treatment led to broader suppression of multiple plakins, including *Ppl*, *Evpl*, and *Dst*. These differences may reflect species-specific immune responses or distinct cytokine environments; the IMQ model primarily activates the IL-17/IL-23 axis and lacks the chronic inflammation and complex immune milieu characteristic of human disease (van der Fits et al., 2009; Swindell et al., 2017). While the IMQ model supports the relevance of EPPK1 loss in inflammatory skin conditions, these limitations underscore the need for more refined models, such as Eppk1-deficient mice crossed with genetic models of psoriasis, to fully dissect the functional role of Eppk1 in psoriasis *in vivo*.

Despite its downregulation in psoriasis, the contribution of EPPK1 to epidermal homeostasis appears to be context- and species-dependent. Although mice lacking Eppk1 do not show overt skin pathology under baseline conditions, several studies suggest that EPPK1 becomes functionally important during stress or injury. For example, *Eppk1* ^*−/−*^ mice show accelerated keratinocyte migration during wound healing (Goto et al., 2006), and Eppk1-deficient liver and pancreas tissues exhibit stress-induced defects (Wögenstein et al., 2014; Szabo et al., 2015). Although our study did not directly assess keratin filament architecture, several previous studies strongly support a role for EPPK1 in cytoskeletal organization under stress. EPPK1 colocalizes with keratins 5, 6, 10, and 17 during wound healing (Ishikawa et al., 2010), indicating a role in keratin network organization under mechanical stress. Mice lacking Eppk1 exhibit thinner keratin filaments, suggesting compromised structural resilience, an effect that may also contribute to impaired barrier function in psoriatic skin (Elango et al., 2018; Cohen et al., 2024). Recent work further supports a stress-responsive function for EPPK1. Under elevated cytoplasmic calcium levels, EPPK1 binds to keratin filaments and stabilizes them by reducing filament dynamics (Ratajczyk et al., 2022). In resting cells, however, EPPK1 remains diffusely cytoplasmic. This dynamic behavior suggests that EPPK1 reinforces cytoskeletal networks specifically during periods of stress or inflammation, a mechanism that may be disrupted in psoriatic skin due to its downregulation.

In summary, our findings highlight a previously underappreciated role for EPPK1 in maintaining epidermal integrity under inflammatory stress. We identify IFN-γ as a key upstream regulator of EPPK1 in psoriatic lesions and propose that *EPPK1* downregulation contributes to disrupted differentiation and weakened barrier function in psoriasis. These results position EPPK1 as potential modulator of epithelial resilience in inflammatory skin diseases. Future research exploring how inflammatory signals intersect with cytoskeletal remodeling could open new therapeutic avenues aimed at reinforcing barrier function in psoriasis.

## Data Availability

The datasets presented in this study can be found in online repositories. The names of the repository/repositories and accession number(s) can be found below: https://www.ncbi.nlm.nih.gov/geo/, GSE300052.
