# Supplementary material for: Epiplakin expression is lost in psoriatic skin lesions and is downregulated by IFN-γ in ex vivo skin cultures
Source: Front Cell Dev Biol. 2025 Jul 17;13:1617737. doi: 10.3389/fcell.2025.1617737 (PMC12310607; doi:10.3389/fcell.2025.1617737)
Supplement: Supplementary file 1 [file DataSheet1.pdf]

Supplementary Material

1 Supplementary figures

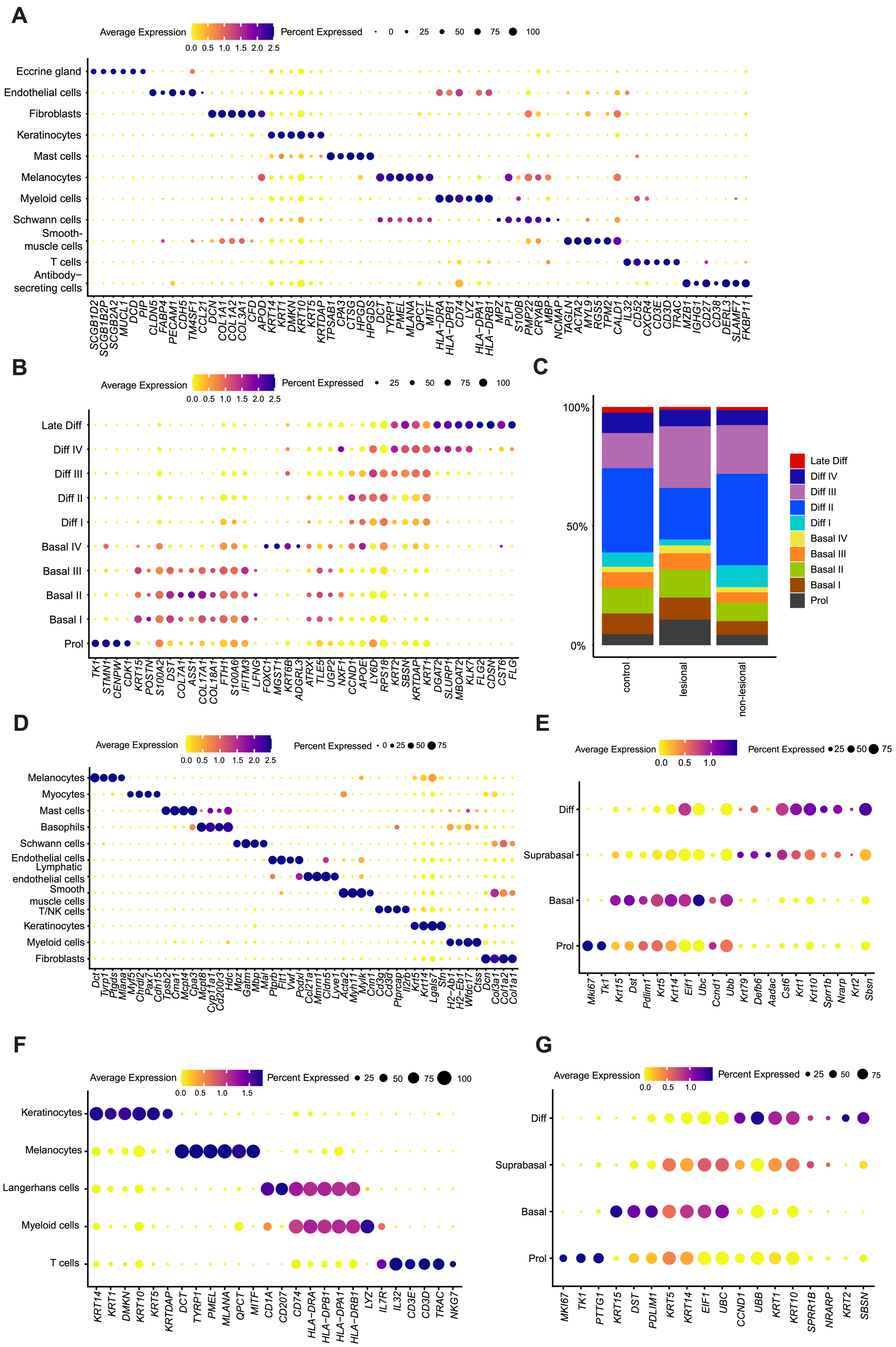

Supplementary Figure S1. Cluster markers and cell proportions

(A) Dot plot of curated cluster markers of the main UMAP of Figure 1, with dot size representing the percentage of cells expressing each marker and color intensity reflecting average gene expression levels. **(B)** Dot plot of the top 4 cluster-specific differentially expressed genes in the keratinocyte subset of Figure 1, ranked by adjusted p-value. **(C)** Stacked bar chart showing the relative proportion of cell types in the keratinocyte subset, split by condition (control, lesional, non-lesional). **(D)** Dot plot of curated cluster markers of the main UMAP of Figure 2 and **(E)** the keratinocyte subset of Figure 2, with dot size representing the percentage of cells expressing each marker and color intensity reflecting average gene expression levels. **(F)** Dot plot of curated cluster markers of the main UMAP of Figure 3 and **(G)** the keratinocyte subset of Figure 3, with dot size representing the percentage of cells expressing each marker and color intensity reflecting average gene expression levels.

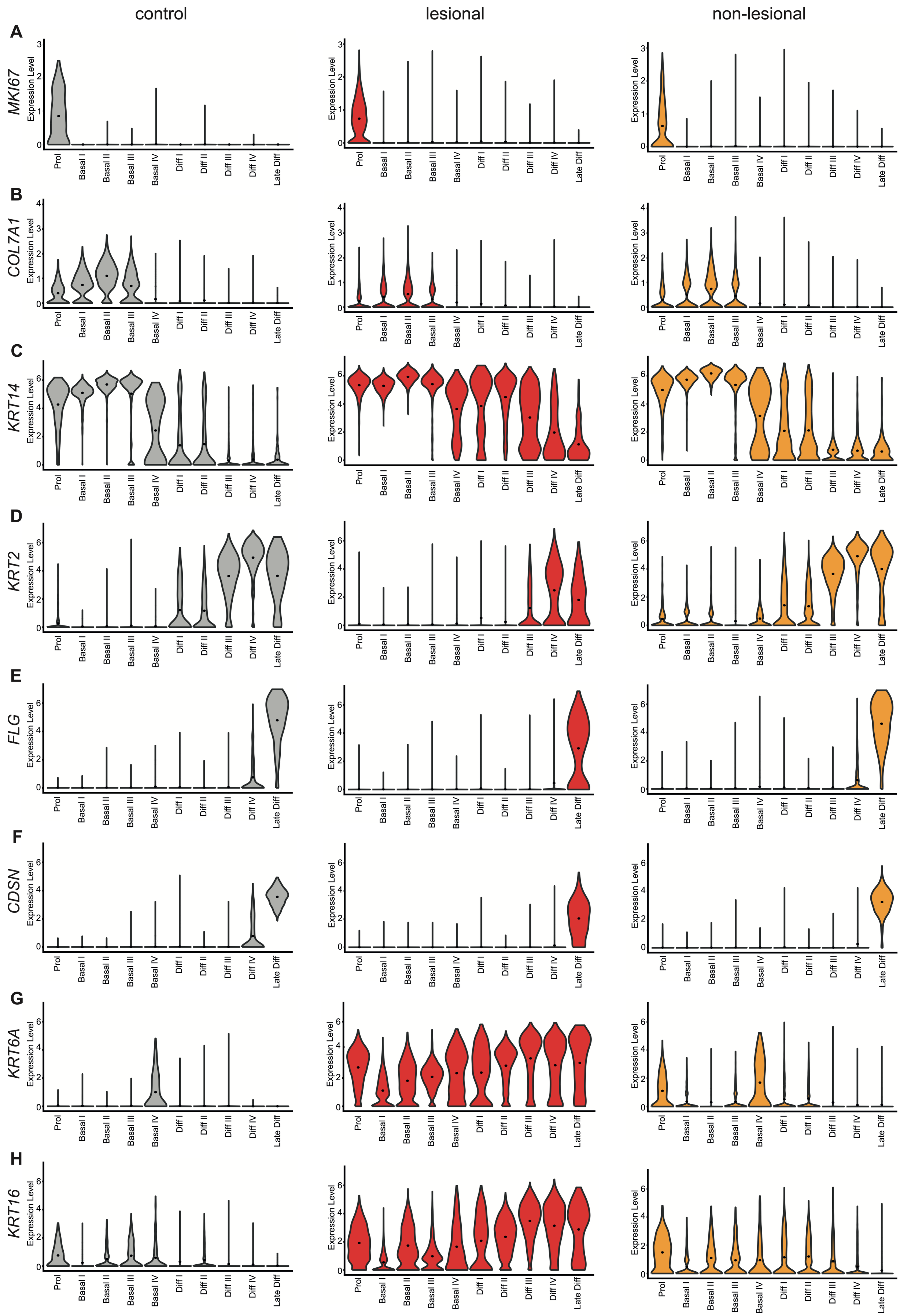
control
lesional
non-lesional
A
MKI67
B
COL7A1
C
KRT14
D
KRT2
E
FLG
F
CDSN
G
KRT6A
H
KRT16
Expression Level
Prol
Basal I
Basal II
Basal III
Basal IV
Diff I
Diff II
Diff III
Diff IV
Late Diff

Supplementary Figure S2. Cluster specific expression of markers for differentiation and psoriasis

Violin plots illustrate the cluster-specific expression levels of **(A)** *MKI67*, **(B)** *COL7A1*, **(C)** *KRT14*, and **(D)** *KRT2* **(E)** *FLG*, **(F)** *CDSN*, **(G)** *KRT6A* and **(H)** *KRT16* in the keratinocyte subset, split by condition (control, lesional, non-lesional). These scRNAseq analysis-derived violin plots depict expression levels in healthy skin (grey), lesional psoriasis (red), and non-lesional psoriasis (orange).

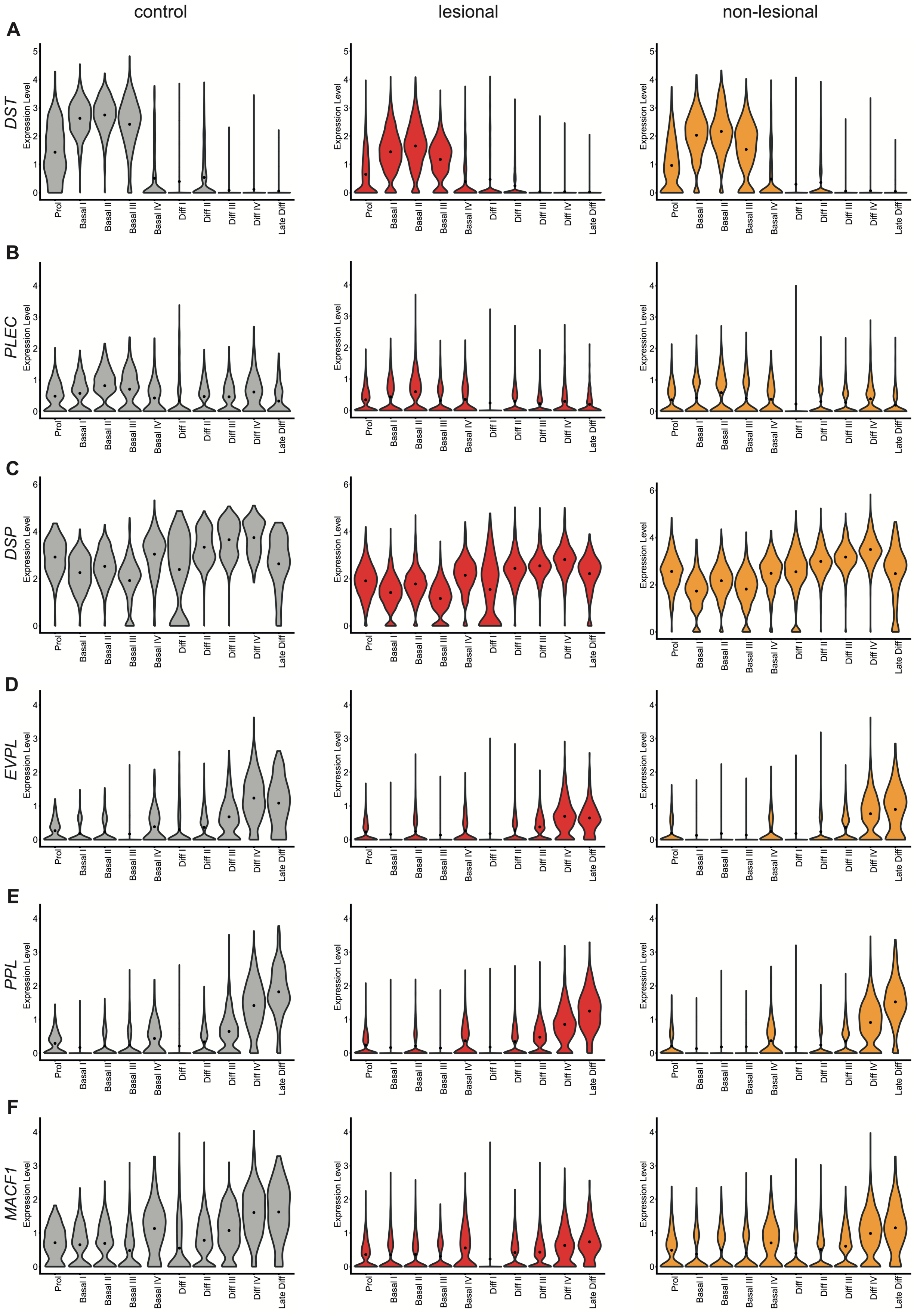
control
lesional
non-lesional
A
B
C
D
E
F
DST
PLEC
DSP
EVPL
PPL
MACF1
Expression Level
Prol
Basal I
Basal II
Basal III
Basal IV
Diff I
Diff II
Diff III
Diff IV
Late Diff

Supplementary Figure S3. Expression of epidermal plakins in keratinocytes from healthy and psoriatic skin.

Cluster specific illustration of epidermal plakin expression levels in the keratinocyte subset split by condition (control, lesional, non-lesional). Violins plots depict expression levels of **(A)** *DST*, **(B)** *PLEC*, **(C)** *DSP* **(D)** *EVPL* **(E)** *PPL* and **(F)** *MACF1* in healthy skin (grey), lesional psoriasis (red), and non-lesional psoriasis (orange) with mean expression indicated by a black dot.

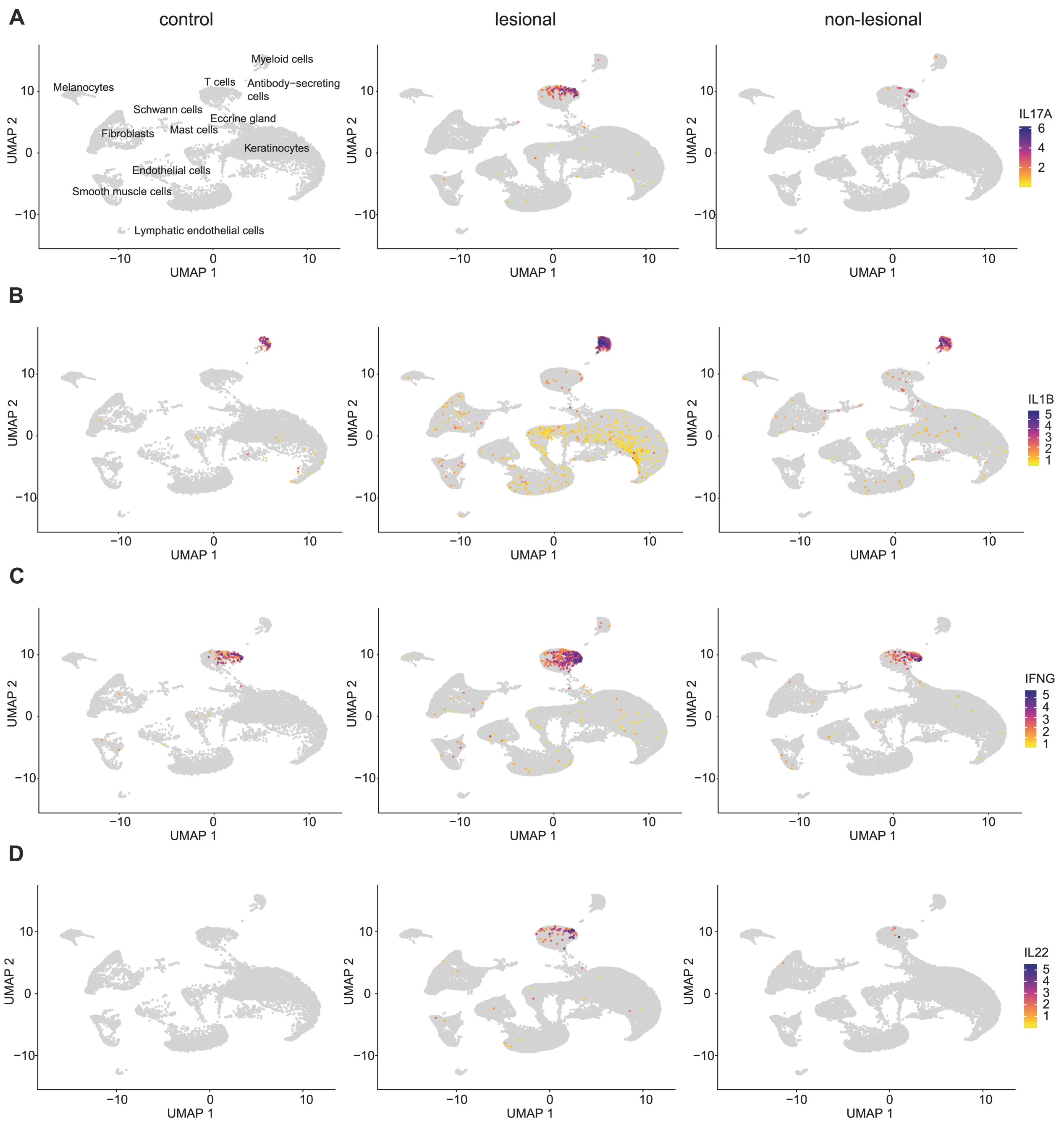

Supplementary Figure S4. Expression levels of cytokines used for ex-vivo treatments

ScCustomize feature plots of the full UMAP illustrating expression levels of cytokines selected for ex-vivo treatment of skin biopsies. **(A)** *IL17A* expression, **(B)** *IL1B* expression, **(C)** *IFNG* and **(D)** IL22 expression, each split by condition into healthy, lesional, and non-lesional psoriasis.

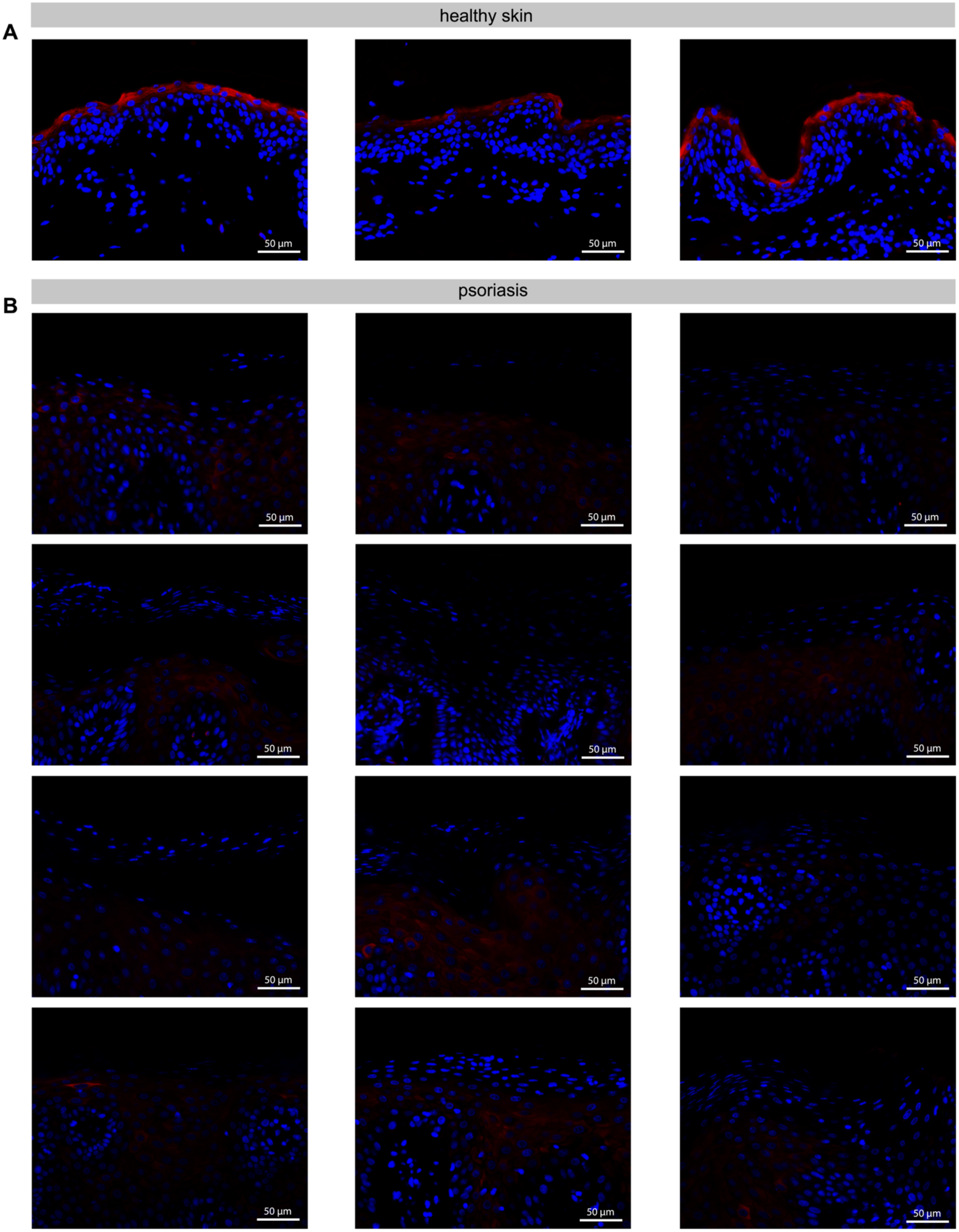
A
healthy skin
B
psoriasis
50 µm

Supplementary Figure S5. Immunostaining of EPPK1 in healthy and psoriatic skin.

(A) Immunofluorescence imaging of skin sections from 3 healthy donors and **(B)** 12 psoriasis skin samples. Images at 40x magnification display DAPI-stained nuclei (blue) and EPPK1 fluorescence (red).

Supplementary Figure S6. Correlation of EPPK1 Corrected Total Cell Fluorescence (CTCF) levels with clinical parameters in psoriasis.

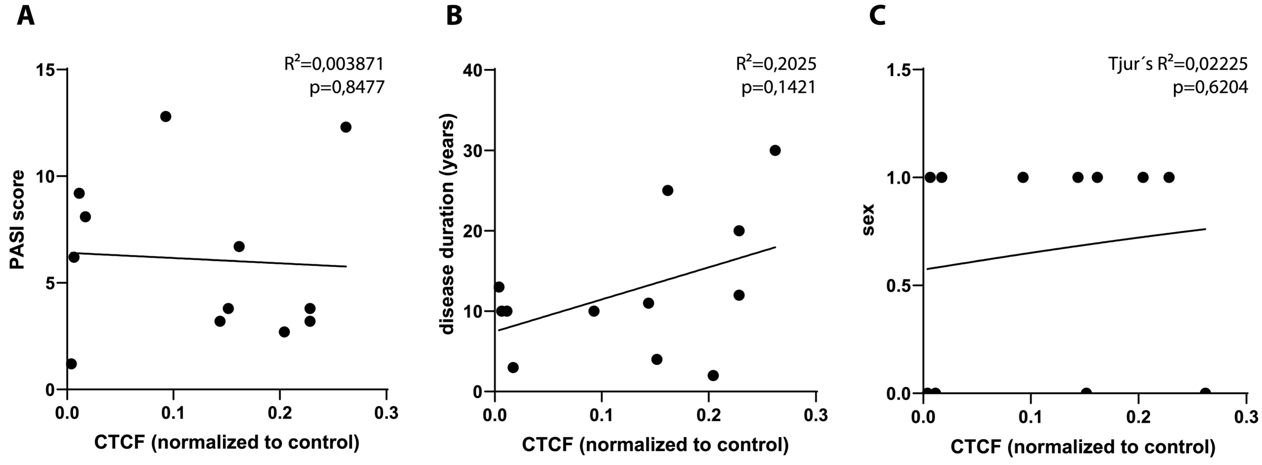

(A) Linear regression analysis of CTCF expression (normalized to mean control skin) and PASI score in psoriatic patients (n = 10). **(B)** Linear regression analysis of normalized CTCF expression and disease duration (in years). **(C)** Logistic regression analysis of normalized CTCF expression and patient sex (coded as 1 = male, 0 = female). R^2 values (Tjur's R^2 for logistic regression) and p-values are indicated. No statistically significant associations were observed.

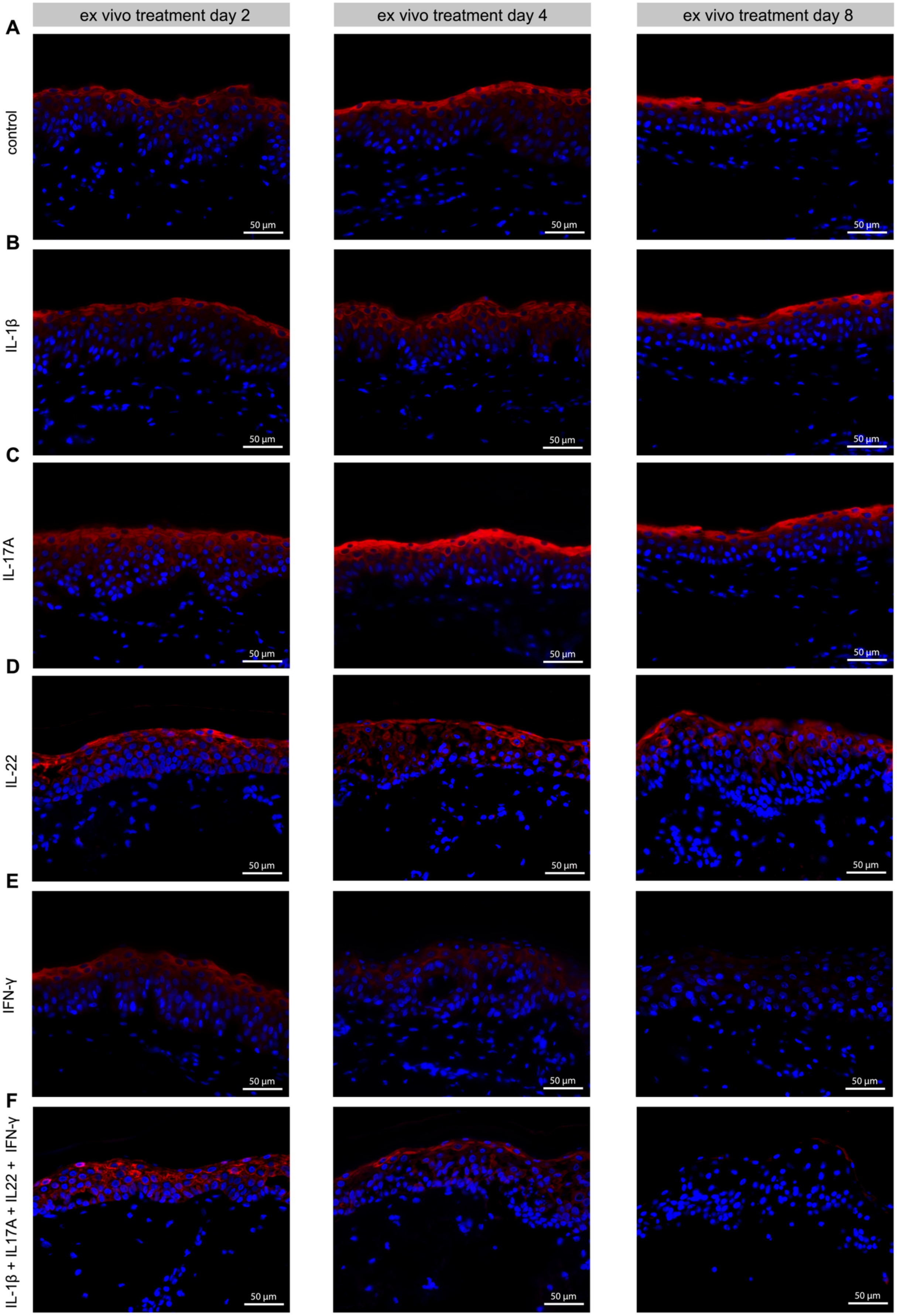
ex vivo treatment day 2
ex vivo treatment day 4
ex vivo treatment day 8
A
control
B
IL-1β
C
IL-17A
D
IL-22
E
IFN-γ
F
IL-1β + IL17A + IL22 + IFN-γ
50 µm

Supplementary Figure S7. Immunostaining of EPPK1 in healthy and psoriatic skin.

Immunofluorescence imaging of skin samples ex vivo at different treatment timepoints at 40x magnification with DAPI-stained nuclei (blue) and EPPK1 fluorescence (red). **(A)** Shows untreated skin ex vivo at days 2, 4, and 8. Images of ex vivo treatment with **(B)** IL-1β, **(C)** IL-17A **(D)** IL-22, **(E)** IFN-γ, and **(F)** a combination of IL-1β, IL-17A, IL22 and IFN-γ, taken at days 2, 4, and 8.

2 Supplementary table

sample ID	diagnosis	sex	PASI score	disease duration
		psoriasis		
PS1	psoriasis	m	3.2	11y
PS2	psoriasis	m	3.2	12y
PS3	psoriasis	f	9.2	10y
PS4	psoriasis	m	6.7	25y
PS5	psoriasis	f	1.2	13y
PS6	psoriasis	m	2.7	2y
PS7	psoriasis	m	8.1	3y
PS8	psoriasis	f	12.3	30y
PS9	psoriasis	m	6.2	10y
PS10	psoriasis	f	3.8	4y
PS11	psoriasis	m	3.8	20y
PS12	psoriasis	m	12.8	10 y
		healthy skin		
HS1	healthy	f	0	0
HS2	healthy	m	0	0
HS3	healthy	f	0	0
HS4	healthy	f	0	0
HS5	healthy	m	0	0
HS6	healthy	m	0	0
HS7	healthy	f	0	0
HS8	healthy	m	0	0
HS9	healthy	m	0	0
HS10	healthy	f	0	0
HS11	healthy	f	0	0

Supplementary Table S1. Immunostaining of EPPK1 in healthy and psoriatic skin.

Overview of individual sample IDs, diagnosis (psoriasis or healthy), sex, Psoriasis Area and Severity Index (PASI) score, and disease duration (in years) for all study participants. PASI scores and disease duration are provided for psoriasis patients only. Healthy control samples were obtained from individuals who had no clinical signs of psoriasis or other inflammatory skin conditions, undergoing elective plastic surgery procedures such as abdominoplasty.
